# The AIRE gene and susceptibility to rheumatoid arthritis in the Spanish population

**DOI:** 10.1186/1479-5876-9-S2-P40

**Published:** 2011-11-23

**Authors:** Belen Torres, Jose Raul García-Lozano, Marco Antonio Montes-Cano, Alicia García, Antonio Núñez-Roldán, Maria Francisca González-Escribano

**Affiliations:** 1 Servicio de Inmunología, HU Virgen del Rocío/IBIS, Sevilla, Spain

## Introduction

AIRE is a transcriptional regulator that plays a functional role in thymocyte education and negative selection by controlling the expression of peripheral antigens in thymus. This gene has been associated with the susceptibility to rheumatoid arthritis (RA) in a recent GWAS carried out in the Japanese population; nevertheless, no association has been described in Caucasian populations.

## Objective

To investigate whether this gene is involved in the susceptibility to RA in the Spanish population.

## Subject and method

A total of 300 Spanish patients fulfilling the ACR criteria for RA and 373 ethnically matched controls were included. Genotyping of 5 Tag SNPs in AIRE: rs878081 (C/T), rs1003854 (T/C), rs933150 (G/A), rs2256817 (G/A) and rs1800522 (T/C) was performed using TaqMan probe assays. Haploview version 4.2 was used for haplotype frequency estimation and the chi-square test to compare allelic distributions.

## Results

The rs878081 was the only SNP found associated with the susceptibility to RA in our cohort (rs878081C 0.79 in patients vs. 0.72 in controls, p=0.002, OR=1.48 95% CI 1.14-1.92). Contrary to the rest of the tag SNPs studied in the present work, the rs878081 has been not included in any RA GWAS. This SNP is located at only 876 bp of the rs2075876 which has been found associated to RA in the Japanese population. Although both SNPs are in linkage disequilibrium, their minoritary allele frequencies are different in Japanese and Caucasian populations (rs878081T: JPT, 0.151 vs. CEU, 0.357 and rs2075876A: JPT, 0.386 vs. CEU, 0.133). Moreover, the risk allele in our study (rs878081C) and the risk allele in the Japanese population study (rs2075876A) are found together in one haplotype in the JPT population (frequency 0.386) and in four haplotypes in the CEU population (accumulated frequency 0.138).

## Conclusion

AIRE could be a risk factor for RA also in Caucasian populations but tag SNPs useful to detect this association may be different in both populations.

